# Differences in BMI *z*-Scores between Offspring of Smoking and Nonsmoking Mothers: A Longitudinal Study of German Children from Birth through 14 Years of Age

**DOI:** 10.1289/ehp.1307139

**Published:** 2014-04-04

**Authors:** Christina Riedel, Nora Fenske, Manfred J. Müller, Sandra Plachta-Danielzik, Thomas Keil, Linus Grabenhenrich, Rüdiger von Kries

**Affiliations:** 1 Institute of Social Paediatrics and Adolescent Medicine, and; 2 Department of Statistics, Ludwig-Maximilians University of Munich, Munich, Germany; 3 Institute of Human Nutrition and Food Science, Christian-Albrechts-University of Kiel, Kiel, Germany; 4 Institute of Social Medicine, Epidemiology and Health Economics, Charité University Medical Center, Berlin, Germany

## Abstract

Background: Children of mothers who smoked during pregnancy have a lower birth weight but have a higher chance to become overweight during childhood.

Objectives: We followed children longitudinally to assess the age when higher body mass index (BMI) *z*-scores became evident in the children of mothers who smoked during pregnancy, and to evaluate the trajectory of changes until adolescence.

Methods: We pooled data from two German cohort studies that included repeated anthropometric measurements until 14 years of age and information on smoking during pregnancy and other risk factors for overweight. We used longitudinal quantile regression to estimate age- and sex-specific associations between maternal smoking and the 10th, 25th, 50th, 75th, and 90th quantiles of the BMI *z*-score distribution in study participants from birth through 14 years of age, adjusted for potential confounders. We used additive mixed models to estimate associations with mean BMI *z*-scores.

Results: Mean and median (50th quantile) BMI *z*-scores at birth were smaller in the children of mothers who smoked during pregnancy compared with children of nonsmoking mothers, but BMI *z*-scores were significantly associated with maternal smoking beginning at the age of 4–5 years, and differences increased over time. For example, the difference in the median BMI *z*-score between the daughters of smokers versus nonsmokers was 0.12 (95% CI: 0.01, 0.21) at 5 years, and 0.30 (95% CI: 0.08, 0.39) at 14 years of age. For lower BMI *z*-score quantiles, the association with smoking was more pronounced in girls, whereas in boys the association was more pronounced for higher BMI *z*-score quantiles.

Conclusions: A clear difference in BMI *z*-score (mean and median) between children of smoking and nonsmoking mothers emerged at 4–5 years of age. The shape and size of age-specific effect estimates for maternal smoking during pregnancy varied by age and sex across the BMI *z*-score distribution.

Citation: Riedel C, Fenske N, Müller MJ, Plachta-Danielzik S, Keil T, Grabenhenrich L, von Kries R. 2014. Differences in BMI *z*-scores between offspring of smoking and nonsmoking mothers: a longitudinal study of German children from birth through 14 years of age. Environ Health Perspect 122:761–767; http://dx.doi.org/10.1289/ehp.1307139

## Introduction

The association of maternal smoking in pregnancy and low birth weight was established several decades ago (Simpson 1957) and is believed to be attributable to intrauterine growth retardation and shortened gestation (Wang et al. 2002). Surprisingly, a number of observational studies in the late 1990s suggested that children of mothers who smoked have a higher body mass index (BMI) later in life and implicitly a higher prevalence of overweight (Fried et al. 1999; Vik et al. 1996; von Kries et al. 1999). This has been confirmed in two meta-analyses of observational studies in populations 3–33 years of age; these studies reported odds ratios of approximately 1.5 for overweight in the children of smoking mothers, though neither meta-analysis addressed age-specific effects (Ino 2010; Oken et al. 2008).

Various aspects of the life-course effect of maternal smoking in pregnancy are not well understood. Some found positive associations (Apfelbacher et al. 2008; Braun et al. 2010; Durmus et al. 2011; Matijasevich et al. 2011; Suzuki et al. 2013), whereas others found no association (Fried et al. 1999; Howe et al. 2012) even if the power was high enough (Harris et al. 2013). Studies in older children have reported a higher prevalence of overweight/higher BMI values in children of smoking mothers for both sexes (Fried et al. 1999; Howe et al. 2012; Power and Jefferis 2002; Salsberry and Reagan 2005; von Kries et al. 2002) or some in boys only (Suzuki et al. 2011, 2012). Crucial questions still remain unanswered: When does a higher BMI in children of smoking mothers emerge? Does the association increase with age? Is the increase in BMI constant over the entire distribution, or does the association differ at the upper tail of the distribution?

We addressed these questions by pooling data from two German cohorts with repeated BMI measurements between birth and 14 years of age and information on maternal smoking during pregnancy and various potential confounders. Potential age-specific effects of maternal smoking during pregnancy across different parts of the BMI distribution were estimated using longitudinal quantile regression, an innovative statistical approach (Fenske et al. 2013).

## Methods

*Study population and data sources*. In Northern Germany, the Kiel Obesity Prevention Study (KOPS), a cluster randomized intervention study, has been performed between 1996 and 2001 by the Institute of Human Nutrition and Food Science of the Christian-Albrechts-University of Kiel in the context of the school entry health examination (SEH; 12,254 children participated in the SEHs during these years) (Plachta-Danielzik et al. 2012b). From these districts in Kiel, 54.6% of the children were randomly chosen and contacted during the recruitment period; among those, 4,997 children (74.7%) agreed to participate in the study (see Supplemental Material, Figure S1) (Plachta-Danielzik et al. 2011). This cohort was representative of all children in Kiel attending the SEH in the recruitment period, as shown by a nonresponse analysis (Plachta-Danielzik et al. 2008). Follow-up information was collected during examinations performed in the school setting, including one examination when the children were in the 4th grade (conducted in 2000–2005, *n* = 4,487), and a second when the children were in the 8th grade (during 2004–2010, *n* = 6,263) (Plachta-Danielzik et al. 2012b). Because of privacy policy, KOPS was not allowed to directly follow-up the children from the SEH; therefore, a pseudonymized study code was used to allow tracking of 1,671 at the 4th and 748 at the 8th grade of the original population. Of these 748 children, 161 children took part in a school intervention program and were excluded. The anthropometric measurements of height and weight were taken by trained nutritionists or collected from the baby check-up booklets (a document given to all parents at birth in which the medical examination results of the child are documented for the first 10 years of life). A self-administered questionnaire with questions on family characteristics and their body compositions was handed out to parents, to be returned by mail. Data on *n* = 330 children with information on weight and height measurements at 0 (birth), 6 (school entry), 10 (4th grade), and 14 (8th grade) years, maternal smoking during pregnancy, and various potential confounders were available.

The second data source was the German Multicenter Allergy Study (MAS) that was launched in 1990. This longitudinal birth cohort study was initiated to investigate the natural course of atopy-related traits in early childhood (Bergmann et al. 1994; Karaolis-Danckert et al. 2008). In six obstetric departments in five German cities (Berlin, Düsseldorf, Freiburg, Mainz, Munich), a questionnaire on atopic diseases was distributed to parents of 7,609 infants who were born in 1990, with a response rate of 79%. The 1,314 healthy mature infants included in the study do not represent a random sample: 499 with a high risk for atopy were included by default, and 815 were selected at random from those children with no risk for atopy (Bergmann et al. 1994; Illi et al. 2006). They were followed up at 1, 3, 6, 12, 18, and 24 months of age, and then annually until the age of 20 years. Four hundred fifty-four (34.6%) of the enrolled children attended all 17 follow-ups, and 721 (54.9%) were examined at 13 years of age. Data on *n* = 719 children with information for the time periods of 0, 0.5, 1, 2, 3, 4, 5, 6, 7, 10, and 13 years were available for the weight and height measurements, maternal smoking during pregnancy, and potential confounders.

Both cohort studies had obtained ethical approval by the respective local ethics committees. This approval included anonymous data analyses beyond the primary scope of the studies.

*Outcome and explanatory variables*. We estimated associations with the BMI *z*-score, defined according to World Health Organization (WHO) guidelines [WHO Child Growth Standards (0–5 years) (WHO Multicentre Growth Reference Study Group 2006) and WHO Reference 2007 (5–19 years) (de Onis et al. 2007)], including differences from the mean and from the 90th, 75th, 50th, 25th, and 10th quantiles of the BMI *z*-score distribution in the study population.

The main explanatory variable was maternal smoking during pregnancy, defined as a binary indicator reflecting any maternal smoking during pregnancy. To adjust for potential confounding in our model, we included maternal weight status at 6 (KOPS) and 10 years of age (MAS) [normal weight (BMI < 25 kg/m^2^), overweight (25 kg/m^2^ ≤ BMI < 30 kg/m^2^), or obese (BMI ≥ 30 kg/m^2^)]; highest maternal education when the child was 6 years of age (KOPS) or a half-year (MAS) (≤ 9, 10–12, and ≥ 13 years of school education); classification of birth weight for gestational age [small for gestational age (weight < 10th percentile according to German reference percentiles) (Voigt et al. 1996), appropriate for gestational age (weight between 10th and 90th percentile), or large for gestational age (weight > 90th percentile)]; preterm delivery (< 37 versus ≥ 37 weeks of gestation); breastfeeding defined as any breastfeeding after birth (yes vs. no); paternal smoking when the child was 6 years (KOPS) and 5 years of age (MAS) (yes vs. no). Unfortunately, maternal prepregnancy weight was not ascertained. The earliest available maternal weight was at 6 or 10 years in these cohorts, and was thus used in this analysis. Similarly, the earliest available maternal education data were collected at the age of 6 years or a half-year, respectively, and the earliest paternal smoking data was collected at the age of 6 or 5 years.

*Statistical analysis*. To test for structural differences between KOPS and MAS, we used Student’s *t*-tests for continuous variables and Fisher’s exact test for categorical variables. Local quantile regression (Yu and Jones 1998) was used to generate unadjusted BMI *z*-score quantile curves (for the 10th, 50th, and 90th quantiles) by age, sex, and maternal smoking.

We used longitudinal quantile regression based on boosting estimation (Fenske et al. 2013) because this method allowed us to simultaneously investigate our three research questions. We also estimated additive mixed models (AMMs) for the mean BMI *z*-score (Fahrmeir et al. 2013), to allow for a comparison with an established approach that has previously been applied to obesity data (Suzuki et al. 2011, 2012).

Quantile regression is a distribution-free approach to estimate effects of explanatory variables on quantiles of the BMI *z*-score distribution. The use of quantile regression allowed us to examine whether the association between smoking and BMI *z*-score is constant over the entire distribution (resulting in an upward shift of the entire distribution from the median value, without any change in the shape of the distribution) or variable, such that the estimated effect of smoking on the upper tail of the BMI distribution (i.e., at the 75th and 90th quantiles) differs from the estimated effect at the lower tail (the 10th and 25th quantiles) or median (50th percentile) of the distribution.

Compared with conventional linear quantile regression (Koenker 2005), the novel approach of additive quantile mixed models (AQMMs) offers additional flexibility in the model predictor. To estimate age-varying effects of maternal smoking during pregnancy on BMI *z*-scores, we included a product interaction term for age and maternal smoking in all models. To account for differences between the MAS and KOPS study populations, we included an additional interaction term for age and study. The potentially nonlinear shapes of these age-varying effects were estimated by P-splines with 20 knots (Eilers and Marx 1996). We adjusted all models for maternal weight status, maternal education, classification of birth weight for gestational age, preterm delivery, breastfeeding, and paternal smoking. To account for intraindividual correlation between repeated measurements typically occurring in longitudinal data, we included individual-specific intercepts and slopes (by age) in the additive predictor. Because some studies reported sex-specific differences (Fried et al. 1999; Howe et al. 2012; Suzuki et al. 2011, 2012), we stratified all analyses by sex. When using AMMs to estimate differences for the population mean, we modeled the same predictors as for AQMMs.

Model estimation for AQMMs was based on boosting and was conducted separately for the previously defined quantiles; this procedure was repeated on 100 subsamples on respectively two-thirds of the full data set to construct 95% CIs for the estimated effects (age-specific 2.5th and 97.5th quantiles of the empirical distribution obtained from 100 subsamples). The presented “best estimate” is the estimate on the complete dataset.

Additional sensitivity analyses were performed to consider further potential confounding variables that were available either in MAS or KOPS data: *a*) early adiposity rebound (AR) (< 5.5 years vs. ≥ 5.5 years). [The AR is the age at which the BMI rises again after its decrease around the age of 1 year; in these data the MAS study provided annual weight measurements. We defined early adiposity according to Rolland-Cachera et al. (1984): age < 5.5 years]; *b*) weight gain during the first year of life (kilograms); *c*) televison consumption at 6 years of age (> 1 hr/day vs. ≤ 1 hr/day); *d*) physical activity in a sports club at 6 years of age (> 2 hr/day vs. ≤ 2 hr/day).

All analyses were carried out with the statistical software R and the add-on packages mboost and gamm4 (http://www.r-project.org/foundation/).

## Results

The proportion of children whose mothers smoked during pregnancy was identical in both data sets, with 20.9% of smoking mothers in both KOPS and MAS (Table 1). There were significant differences between both cohorts regarding sex, maternal education, classification of birth weight for gestational age, breastfeeding, and paternal smoking. However, birth weight and length as well as BMI *z*-scores at 6 and 10 years of age did not significantly differ between studies.

**Table 1:** Comparison of population characteristics between the two German cohorts [*n* (%) or mean ± SD]

Variable	KOPS	MAS	*p*-Value
No. of children	330	781
No. of observations	1,320	7,228
Parental characteristics
Maternal smoking during pregnancy
Yes	69 (20.9)	150 (20.9)
No	261 (79.1)	569 (79.1)	1.00
Maternal weight status
Normal weight	244 (73.9)	549 (76.4)
Overweight	64 (19.4)	127 (17.7)
Obese	22 (6.7)	43 (6.0)	0.684
Highest maternal education (years)
≤ 9	44 (13.3)	197 (27.4)
10–12	106 (32.1)	237 (33.0)
≥ 13	180 (54.5)	285 (39.6)	< 0.001
Paternal smoking
Yes	126 (38.2)	176 (24.5)
No	204 (61.8)	543 (75.5)	< 0.001
Child characteristics
Sex
Female	177 (53.6)	332 (46.2)
Male	153 (46.4)	387 (53.8)	0.028
Classification of birth weight for gestational age
Small	30 (9.09)	93 (12.9)
Average	261 (79.1)	579 (80.5)
Large	39 (11.8)	47 (6.5)	0.006
Preterm delivery (< 37 weeks)
Yes	16 (4.8)	18 (2.5)
No	314 (95.2)	701 (97.5)	0.059
Breastfeeding at any time after birth
Yes	277 (83.9)	676 (94)
No	53 (16.1)	43 (6)	< 0.001
Birth weight (g)	3,440 ± 559	3,422 ± 470	0.604
Birth length (cm)	51.6 ± 2.9	51.4 ± 2.3	0.219
BMI *z*-score at 6 years	–0.01 ± 1.0	0.11 ± 1.0	0.106
BMI *z*-score at 10 years	0.24 ± 1.1	0.36 ± 1.2	0.185
BMI > +1 SD^*a*^ at 6 years (%)	10.6	17.8	0.044
BMI > +1 SD^*a*^ at 10 years (%)	22.2	27.4	0.148
BMI > +2 SD^*a*^ at 6 years (%)	2.1	4.9	0.176
BMI > +2 SD^*a*^ at 10 years (%)	3.0	9.9	< 0.001
Variables for the sensitivity analyses^*b*^
Television consumption
> 1 hr	104 (42.6)	—
≤ 1 hr	140 (57.4)	—
Physical activity in a sports club
> 2 hr	108 (45.0)	—
≤ 2 hr	132 (55.0)	—
Weight gain during the first year of life (kg)	—	12.7 (2.0)
Early adiposity rebound (≤ 5.5 years)
Yes	—	174 (31.8)
No	—	374 (68.2)
—, not available in the respective cohort. ^***a***^Overweight (BMI > +1 SD) is equivalent to BMI 25 kg/m^2^ at 19 years, and obesity (BMI > +2 SD) is equivalent to BMI 30 kg/m^2^ at 19 years (de Onis et al. 2007). ^***b***^Difference in number of cases compared with those in the upper part of the table can be explained by an increasing number of missing values.			

To assess whether the two data sets can be combined, we additionally evaluated potential differences in the BMI *z*-score increase by age in the respective cohorts (similar increments). Scatterplots showed a similar distribution of the BMI *z*-score values around the regression line of BMI *z*-score by age (see Supplemental Material, Figure S2), and the 95% CIs of the increment in BMI *z*-score per year overlapped (MAS: 0.032; 95% CI: 0.025, 0.038; and KOPS: 0.046; 95% CI: 0.036, 0.057). To assess the consistency of the association of potential confounders with the age-dependent BMI *z*-score values, we tested for potential effect modification of the association of the potential confounders considered in the final data set and BMI *z*-score by study by modeling interaction terms between study (MAS or KOPS) and the following confounders: sex, maternal weight status, maternal education, classification of birth weight, breastfeeding, preterm delivery, and paternal smoking. Interaction terms were not statistically significant except for the variables small for gestational age and preterm delivery (see Supplemental Material, Table S1). For both variables, positive associations with BMI *z*-scores were greater for the MAS study, possibly because only term or near-term children were recruited for MAS, in contrast with KOPS, where all children were recruited regardless of their gestational age.

Potential differences in risk factors for childhood obesity between smoking and nonsmoking mothers during pregnancy are shown in Table 2. Smoking mothers were more likely to be less educated than nonsmoking mothers. The children of smoking mothers were less likely to be breastfed and more likely to have a smoking father, and had a significantly lower mean birth weight and length (accounting for more children born small for gestational age) than the children of nonsmoking mothers. Mean BMI *z*-scores at 6 and 10 years of age were higher in the children of mothers who smoked during pregnancy.

**Table 2:** Overview of variables contained in the final dataset with *n* = 1,049 children by maternal smoking during pregnancy (yes vs. no) [*n* (%) or mean ± SD].

Variable	Maternal smoking during pregnancy	No maternal smoking during pregnancy	*p*-Value
No. of children	219	830
No. of observations	1,755	6,793
Parental characteristics
Maternal weight status
Normal weight	162 (74.0)	631 (76.0)
Overweight	40 (18.3)	151 (18.2)
Obese	17 (7.8)	48 (5.8)	0.525
Highest maternal education (years)
≤ 9	89 (40.6)	152 (18.3)
10–12	70 (32.0)	273 (32.9)
≥ 13	60 (27.4)	405 (48.8)	< 0.001
Paternal smoking
Yes	95 (43.4)	207 (24.9)
No	124 (56.6)	623 (75.1)	< 0.001
Child characteristics
Sex
Female	113 (51.6)	396 (47.7)
Male	106 (48.4)	434 (52.3)	0.324
Classification of birth weight for gestational age
Small	37 (16.9)	86 (10.4)
Average	172 (78.5)	668 (80.5)
Large	10 (4.6)	76 (9.2)	0.004
Preterm delivery
Yes	6 (2.7)	28 (3.4)
No	213 (97.3)	802 (96.6)	0.830
Breastfeeding at any time after birth
Yes	185 (84.5)	768 (92.5)
No	34 (15.5)	62 (7.5)	0.001
Birth weight (g)	3,279 ± 492	3,467 ± 494	< 0.001
Birth length (cm)	50.7 ± 2.4	51.6 ± 2.5	< 0.001
BMI *z*-score at 6 years	0.34 ± 1.0	0.01 ± 1.0	< 0.001
BMI *z*-score at 10 years	0.54 ± 1.2	0.25 ± 1.1	0.020
BMI > +1 SD^*a*^ at 6 years (%)	23.6	14.6	0.011
BMI > +1 SD^*a*^ at 10 years (%)	34.7	23.6	0.007
BMI > +2 SD^*a*^ at 6 years (%)	7.0	3.7	0.083
BMI > +2 SD^*a*^ at 10 years (%)	11.3	6.9	0.088
Variables for the sensitivity analyses^*b*^
Television consumption
> 1 hr	27 (60.0)	77 (38.7)
≤ 1 hr	18 (40.0)	122 (61.3)	0.014
Physical activity in a sports club
> 2 hr	16 (37.2)	92 (46.7)
≤ 2 hr	27 (63.0)	105 (53.3)	0.311
Weight gain during the first year of life (kg)	12.9 (1.7)	12.7 (2.0)	0.283
Early adiposity rebound (≤ 5.5 years)
Yes	38 (35.8)	136 (30.8)
No	68 (64.2)	306 (69.2)	0.492
^***a***^Overweight (BMI > +1 SD) is equivalent to BMI 25 kg/m^2^ at 19 years, and obesity (BMI > +2 SD) is equivalent to BMI 30 kg/m^2^ at 19 years (de Onis et al. 2007). ^***b***^Difference in number of cases compared with those in the upper part of the table can be explained by an increasing number of missing values.			

**Figure 1:**
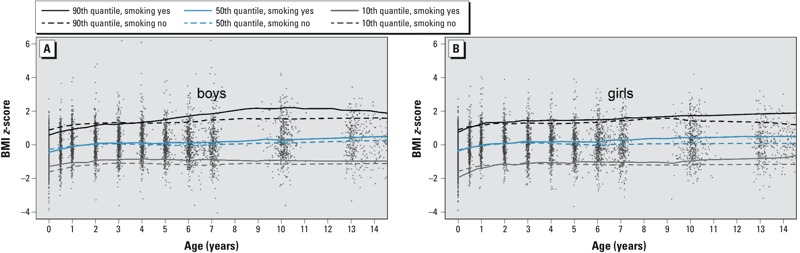
All observations (gray points) of boys (*A*) and girls (*B*) with empirical 10th, 50th, and 90th BMI z-score quantile curves by age and maternal smoking ­during pregnancy.

Figure 1 shows all BMI *z*-scores according to age for all observations, and depicts the (unadjusted) time course of BMI *z*-score quantiles by age, sex, and maternal smoking during pregnancy. In boys (Figure 1A), the 10th BMI *z*-score quantile curve for children of smoking mothers is constantly higher than the curve for children of nonsmoking mothers. Regarding higher quantiles in boys, the curves of BMI *z*-score quantiles for children of smoking mothers were below or equal to the curves in children of nonsmoking mothers up to the age of 4 years, and became progressively higher thereafter. In girls (Figure 1B) of smoking mothers, the 10th BMI *z*-score quantile curve was below that of nonsmoking mothers during the first year of life. Afterward, both curves overlapped up to 5 years of age, when a progressively higher BMI emerged for children of smoking mothers until adolescence. For higher quantiles this difference emerged earlier, at the age of about 2–3 years.

**Figure 2:**
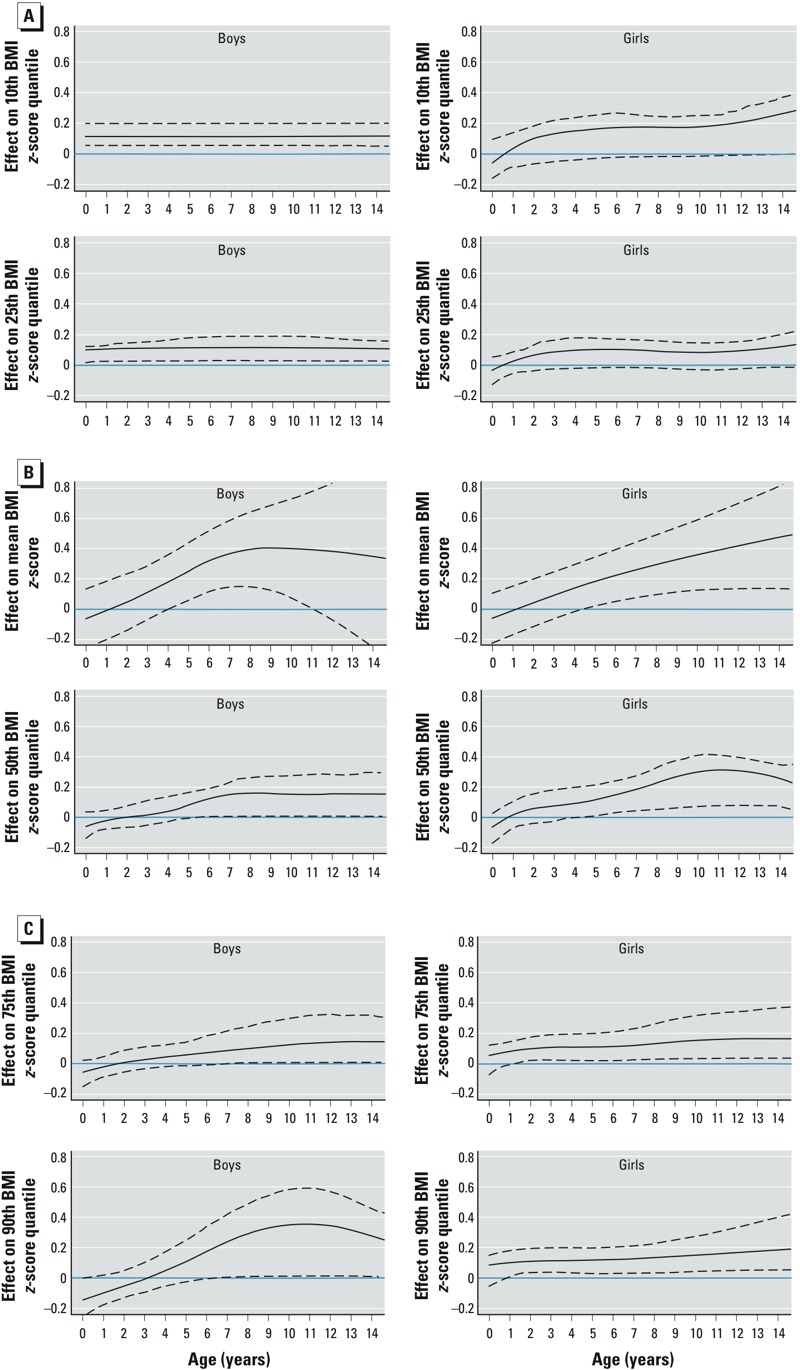
Age-varying effect estimates for maternal smoking during pregnancy (black lines) compared with nonsmoking during pregnancy (blue horizontal line at zero) for boys (left) and girls (right), adjusted by maternal weight status, highest maternal education, classification of birth weight for gestational age, preterm delivery, breastfeeding, paternal smoking, and by the interaction term of age and study. (*A*) 10th (upper row) and 25th (lower row) BMI *z*-score quantile resulting from AQMMs; (*B*) mean BMI *z*-score (upper row) resulting from AMMs, and 50th BMI *z*-score quantile (lower row) resulting from AQMMs; (*C*) 75th (upper row) and 90th (lower row) BMI *z*-score quantiles resulting from AQMMs. Results from the AQMMs: black lines = best estimates, dashed lines = 95% CI, based on the 100 subsamples; results from the AMMs: black lines = estimated effect, dashed lines = 95% CI.

The age-dependent adjusted differences between the BMI *z*-scores in boys and girls are depicted in Figure 2 (for underlying values, see Supplemental Material, Table S2). Emergence of higher BMI *z*-scores in children of smoking mothers was defined as the age when the lower limit of the 95% CI for BMI *z*-score difference first exceeds zero. This was considered statistically significant. In boys, the BMI *z*-score for the 10th quantile (Figure 2A) was 0.12 higher in association with maternal smoking versus nonsmoking at all ages. For lower BMI *z*-score quantiles (10th and 25th) in girls, the difference between the children of smokers versus nonsmokers emerges between 4 and 6 years of age, and increases until adolescence for the 10th quantile or remains constant over all ages for the 25th quantile. Similarly, for mean and median BMI *z*-scores in both boys and girls, significantly higher BMI *z*-scores in children of smoking mothers were estimated at 4–5 years of age (Figure 2B). For the 50th BMI *z*-score quantile, the estimated effect of maternal smoking was –0.06 at birth for both sexes, reflecting the child’s lower birth weight compared with children of mothers who did not smoke during pregnancy. However, at 4–5 years of age in boys and girls, BMI *z*-scores were significantly higher in the children of smoking mothers compared with the children of nonsmoking mothers. In girls, the difference increased with age, such that the difference in the median BMI *z*-score increased from 0.12 (95% CI: 0.02, 0.24) at 5 years to 0.30 (95% CI: 0.08, 0.39) at 12.5 years of age, whereas the estimated difference in the mean BMI *z*-score increased linearly through 14 years of age. In boys, the difference in estimated mean and median BMI *z*-scores increased to about 7 years of age only. In the upper tail (90th quantile) of the BMI *z*-score distribution (Figure 2C) differences between the children of smokers versus nonsmokers were more pronounced in boys than in girls.

Among the potential confounders, large for gestational age was associated with significantly higher mean BMI *z*-scores, whereas small for gestational age and preterm delivery were associated with significantly lower mean BMI *z*-scores based on AMM models adjusted for smoking and all other covariates in the final model (see Supplemental Material, Table S3).

Estimated associations between maternal smoking and mean BMI *z*-scores were less precise and somewhat closer to the null when adjusted for covariates available for one study population only (television consumption and physical activity for KOPS; early adiposity rebound and weight gain during the first year of life in MAS) (see Supplemental Material, Table S4 and Figure S3).

## Discussion

Based on differences in mean and median BMI *z*-scores, a positive association between smoking during pregnancy and overweight in children emerged at approximately 4–6 years of age and increased until adolescence. At lower quantiles the association was more pronounced in girls than in boys, whereas for higher quantiles the association was more pronounced and increased to a greater extent over time in boys compared with girls. Some previous studies have compared BMI or BMI *z*-scores in cohorts of children of smoking and nonsmoking mothers in repetitive cross-sectional analyses (Florath et al. 2013; Fried et al. 1999; Power and Jefferis 2002; Vik et al. 1996). The time period varied from birth to 33 years of age, although not all studies considered the life course since birth (Power and Jefferis 2002). In general, results of these studies suggest that the effect of maternal smoking on overweight in children increases with age. A few studies have attempted to model the longitudinal course in children after preschool years (Chen et al. 2006; Haga et al. 2012; Howe et al. 2012; Pryor et al. 2011; Suzuki et al. 2011, 2012). Pryor et al. (2011) and Haga et al. (2012) examined the impact of maternal smoking on predefined BMI accretion patterns in children, whereas our modeling was not based on such predefined patterns. Consistent with our findings, these authors reported that the association between maternal smoking during pregnancy became evident at 4–5 years of age and increased thereafter. Others have used more flexible models to evaluate the association between maternal smoking and weight in children, but effect estimates were limited to differences in mean BMI (Chen and Kelly 2005; Howe et al. 2012; Suzuki et al. 2011) and mean BMI *z*-scores (Suzuki et al. 2011, 2012) up to 10 years of age. In most cases, these studies also reported stronger associations between maternal smoking and child’s weight with increasing age.

Differences by sex also have been reported by several studies, but with equivocal directions: higher effect estimates for boys than for girls (Fried et al. 1999; Power and Jefferis 2002; Suzuki et al. 2011, 2012) or vice versa (Chen et al. 2006; Howe et al. 2012). These equivocal findings might be related to differential effects on different parts of the BMI/BMI *z*-score distribution, pointing to the potential importance of quantile specific analyses.

The main strength of our analysis is a long follow-up from birth until early adolescence, which allowed modeling the BMI *z*-score life course across the BMI *z*-score distribution with adjustment for potential confounders. Therefore, the innovative contribution of our analysis is that it takes the longitudinal data structure into account in a flexible manner, and that it considers percentile-specific effects.

Assessment of maternal smoking during pregnancy was based on maternal self-reporting, which could lead to misclassification. However, Nafstad et al. (1996) demonstrated good consistency between maternal self-reported daily cigarette consumption and cotinine concentration in cord blood, suggesting fair validity of maternal reporting on smoking. Although that study was conducted in a sample with a somewhat higher prevalence of smoking mothers [Oslo cohort (Nafstad et al. 1996): 32.7%; 95% CI: 26.3, 39.6; and our cohort: 20.9%; 95% CI: 18.5, 23.5], this is unlikely to account for a different validity of the maternal reporting on smoking.

A limitation of our data is the lack of information regarding the extent of maternal smoking during pregnancy. Several studies reported evidence of a dose–response effect of the number of cigarettes smoked during pregnancy on the risk of overweight or obesity (Koshy et al. 2011; Montgomery and Ekbom 2002; Wideroe et al. 2003). We had data only on the number of cigarettes per day in the MAS cohort. Of 142 smoking mothers, 109 smoked 1–10 cigarettes/day during pregnancy, and only 33 smoked > 10 cigarettes. Mean BMI *z*-scores did not differ between those of children of heavy- and light-smoking mothers during pregnancy at respective ages (0, 1, 2, 3, 4, 5, 6, 7, 10, and 13 years) (data not shown), but this may have been attributable at least partly to the small number of heavy-smoking mothers. Another limitation is that only a subset of children from the original MAS and KOPS study had sufficient follow-up, outcome, and confounder data to be included in the present analysis, but there were no significant differences between the study samples and the full cohorts with regard to sex, birth weight, birth length, and BMI *z*-scores at different ages (data not shown).

We used additive mixed models and the innovative statistical approach of longitudinal quantile regression to estimate differences according to within-population BMI *z*-score quantiles and simultaneously investigate our three research objectives. A major strength of our approach was the inclusion of an age-varying effect of maternal smoking during pregnancy, which enabled us to identify the age at which the positive association emerges and to estimate nonlinear changes over time.

Although our findings do not provide direct evidence for a causal relation between maternal smoking during pregnancy and increasing BMI differences, they point to some similarities with randomized animal studies on intrauterine nicotine exposure (Gao et al. 2005; Oliveira et al. 2009; Somm et al. 2008). As in these animal studies, the impact of maternal smoking on BMI in the children appeared to increase with age. Changes in the hypothalamic regulation of energy homeostatic resulting in changes in appetite control and energy expenditure might be instrumental (Bruin et al. 2010; Grove et al. 2001; Holloway et al. 2005).

Previous studies have reported that associations with paternal smoking or secondhand smoke during and after pregnancy are similar to (Harris et al. 2013; Howe et al. 2012; Kleiser et al. 2009; Plachta-Danielzik et al. 2012a; von Kries et al. 2008) or stronger than (Apfelbacher et al. 2008; Florath et al. 2013; Raum et al. 2011) associations with maternal smoking during pregnancy, based on mutually adjusted models. Paternal and maternal smoking both may be markers of unmeasured family characteristics, and although adjusting for paternal smoking did not eliminate age-varying associations between maternal smoking during pregnancy and BMI *z*-scores, residual confounding cannot be ruled out as an alternative explanation for our findings.

## Conclusion

Given combined data from two longitudinal cohort study populations, we estimated higher mean and median BMI *z*-scores in the children of mothers who smoked during pregnancy compared with other children, with significant differences emerging at 4–6 years of age and increasing over time. Whether this is a reflection of an epigenetic priming mechanism accounting for progressively increasing effects or residual confounding by an incremental unknown exposure remains unclear.

## Supplemental Material

(705 KB) PDF
