# Supplementary material for: Differences in BMI z-Scores between Offspring of Smoking and Nonsmoking Mothers: A Longitudinal Study of German Children from Birth through 14 Years of Age
Source: Environ Health Perspect. 2014 Apr 4;122(7):761–7. doi: 10.1289/ehp.1307139 (PMC4080541; doi:10.1289/ehp.1307139)
Supplement: (705 KB) PDF [file ehp.1307139.s001.pdf]

Environmental Health Perspectives doi:10.1289/ehp.1307139

Supplemental Material

Differences in BMI *z*-Scores between Offspring of Smoking and Nonsmoking Mothers: A Longitudinal Study of German Children from Birth through 14 Years of Age

Christina Riedel, Nora Fenske, Manfred J. Müller, Sandra Plachta-Danielzik, Thomas Keil, Linus Grabenhenrich, and Rüdiger von Kries

Table of Contents **Page**

Figure S1: Flow chart of sample size of KOPS. 2

Figure S2: Distribution of BMI *z*-score values of both cohorts. 3

Table S1: P-values and t-values of interaction of all variables of the full model and the study variable. 4

Table S2: Estimated effects (Best estimate (95% CI)) and mean (95% CI)) for the age-varying effects of maternal smoking during pregnancy at the ages of 0, 2.5, 5, 7.5, 10, 12.5 and 14 years with quantile regression for the 10th 25th 50th 75th and 90th BMI *z*-score quantiles and mean regression for mean BMI *z*-score. 5

Table S3: Mutually adjusted effects of the categorical potential confounders on the mean BMI *z*-score and BMI *z*-score quantiles obtained from the final models estimating the effect of maternal smoking during pregnancy (AMMs and AQMMs). 6

Table S4: Univariate and multivariate effect estimates β (95%CI) of the potential confounders only available in either MAS (early adiposity rebound, weight gain during the first two years of life) or KOPS (TV consumption, physical activity) on the mean BMI *z*-score. 7

Figure S3: Age-varying effect of maternal smoking during pregnancy compared to non-smoking mothers during pregnancy for boys and girls adjusted by a) TV consumption and physical activity in sports club (KOPS n = 107 boys and 132 girls) and b) early adiposity rebound and weight gain during the first year of life (MAS n = 351 boys and 299 girls) resulting for mean BMI *z*-score values from the additive mixed model. 8

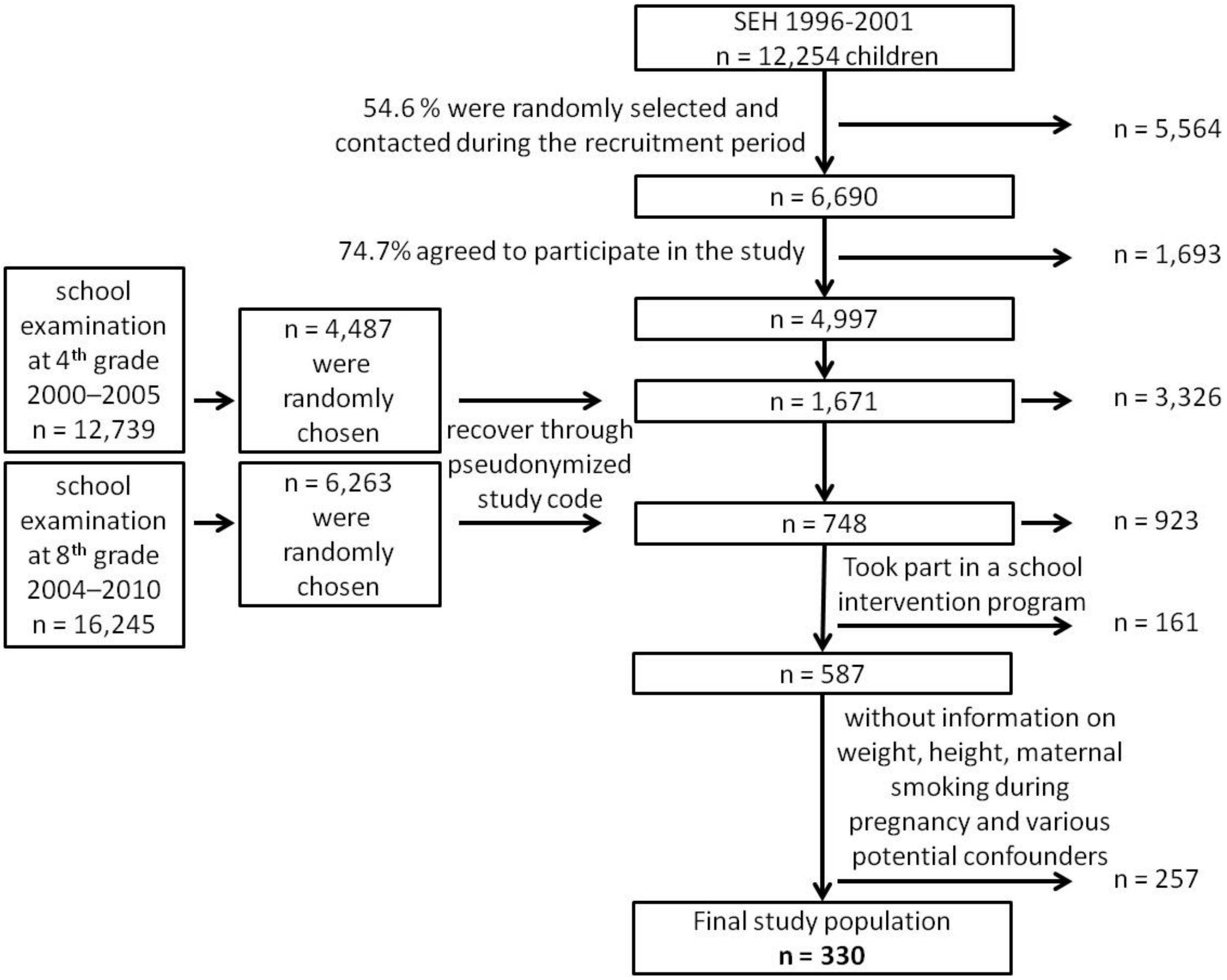

Figure S1: Flow chart of sample size of KOPS.

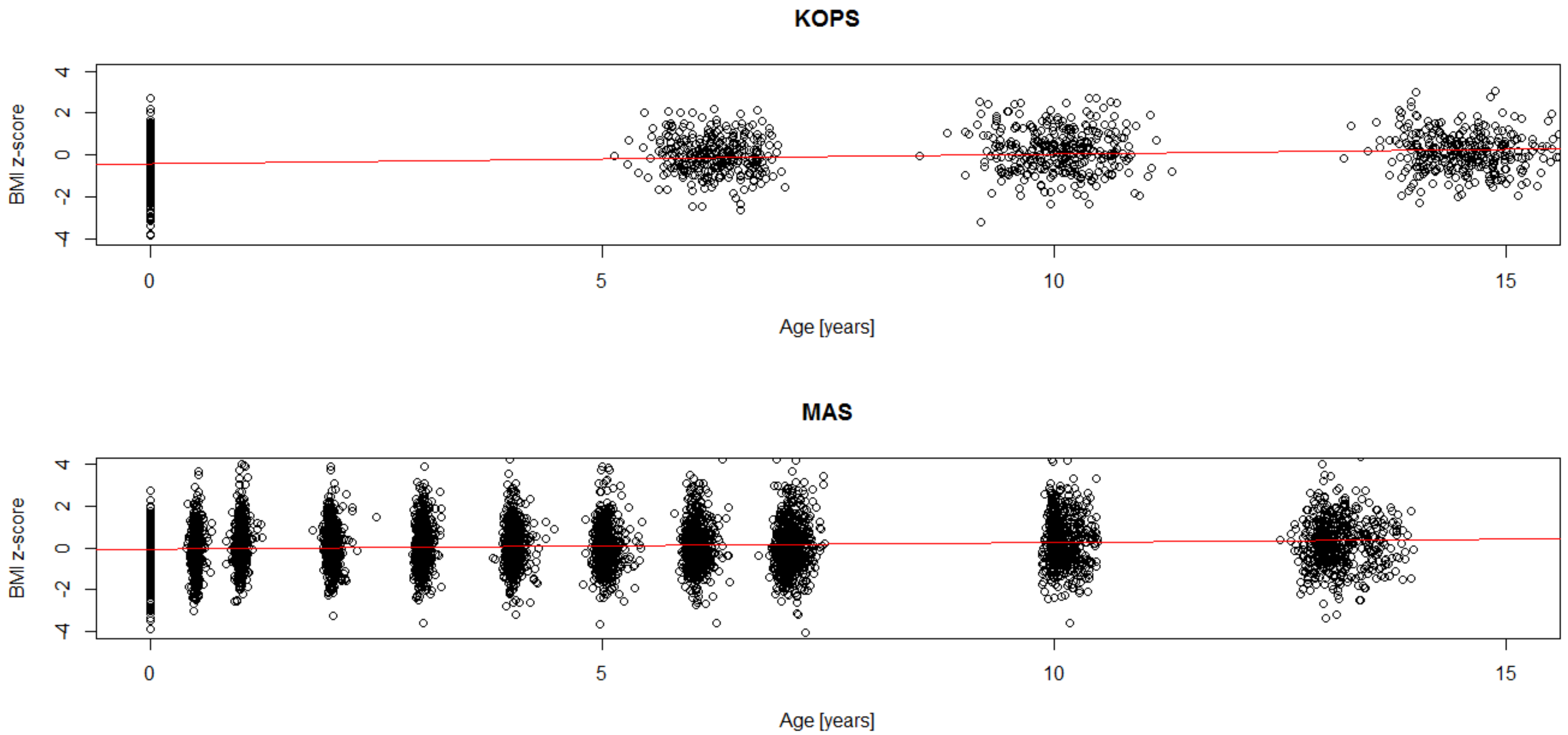

Figure S2: Distribution of BMI *z*-score values of both cohorts. The red line presents the linear regression estimation for BMI *z*-score and age.

Table S1: P-values and t-values of interaction of all variables of the full model and the study variable.

**Interaction variables**	**t-value**	**p-value**
Gender = male & study = MAS	0.128	0.898
Maternal weight status = overweight & study = MAS	0.002	0.998
Maternal weight status = obese & study = MAS	-0.323	0.746
Maximal maternal education = ≤ 9 years of school education & study = MAS	-0.415	0.678
Maximal maternal education = 10-12 years of school education & study = MAS	-1.539	0.124
Large for gestational age & study = MAS	-0.313	0.754
Small for gestational age & study = MAS	2.765	0.006
Breastfeeding & study = MAS	1.296	0.195
Preterm delivery & study = MAS	3.121	0.002
Paternal smoking & study = MAS	-0.649	0.516

Table S2: Estimated effects (Best estimate (95% CI)) and mean (95% CI)) for the age-varying effects of maternal smoking during pregnancy at the ages of 0, 2.5, 5, 7.5, 10, 12.5 and 14 years with quantile regression for the 10th, 25th 50th, 75th and 90th BMI *z*-score quantiles and mean regression for mean BMI *z*-score.

**Model**	**0 years**	**2.5 years**	**5 years**	**7.5 years**	**10 years**	**12.5 years**	**14 years**
10th quantile							
boys	0.12 (0.06,0.20)	0.12 (0.06,0.20)	0.12 (0.06,0.20)	0.12 (0.06,0.20)	0.12 (0.06,0.20)	0.12 (0.06,0.20)	0.12 (0.05,0.20)
girls	-0.06 (-0.16,0.10)	0.12 (-0.06,0.20)	0.17 (-0.03,0.25)	0.18 (-0.02,0.25)	0.18 (-0.01,0.25)	0.22 (0.00,0.31)	0.27 (0.00,0.36)
25th quantile							
boys	0.10 (0.02,0.12)	0.11 (0.03,0.15)	0.11 (0.03,0.18)	0.12 (0.03,0.19)	0.12 (0.03,0.19)	0.11 (0.03,0.17)	0.11 (0.03,0.16)
girls	-0.03 (-0.12,0.05)	0.08 (-0.03,0.15)	0.10 (-0.01,0.18)	0.10 (-0.02,0.16)	0.08 (-0.03,0.14)	0.10 (-0.02,0.16)	0.12 (-0.01,0.20)
50th quantile							
boys	-0.06 (-0.14,0.03)	0.01 (-0.06,0.09)	0.08 (-0.01,0.16)	0.16 (0.01,0.25)	0.15 (0.01,0.27)	0.16 (0.01,0.28)	0.15 (0.01,0.30)
girls	-0.06 (-0.17,0.03)	0.07 (-0.03,0.17)	0.12 (0.01,0.21)	0.21 (0.05,0.29)	0.30(0.07,0.41)	0.30 (0.08,0.39)	0.26 (0.08,0.35)
75th quantile							
boys	-0.05 (-0.15,0.02)	0.02 (-0.04,0.10)	0.06 (-0.01,0.14)	0.09 (0.00,0.23)	0.13 (0.01,0.30)	0.14 (0.01,0.32)	0.15 (0.01,0.32)
girls	0.06 (-0.07,0.12)	0.10 (0.02,0.18)	0.11 (0.02,0.20)	0.13 (0.03,0.24)	0.15 (0.04,0.32)	0.16 (0.04,0.35)	0.16 (0.04,0.37)
90th quantile							
boys	-0.14 (-0.25,0.00)	-0.03 (-0.11,0.07)	0.11 (-0.03,0.25)	0.27 (0.01,0.44)	0.35 (0.01,0.58)	0.33 (0.01,0.56)	0.27 (0.01,0.48)
girls	0.09 (-0.05,0.15)	0.11 (0.04,0.20)	0.12 (0.03,0.20)	0.13 (0.03,0.22)	0.15 (0.04,0.28)	0.17 (0.05,0.35)	0.19 (0.05,0.4)
mean							
boys	-0.06 (-0.26,0.14)	0.08 (-0.10,0.26)	0.25 (0.06,0.44)	0.38 (0.15,0.61)	0.40 (0.08,0.73)	0.38 (-0.06,0.83)	0.35 (-0.24,0.94)
girls	-0.06 (-0.23,0.11)	0.07 (-0.08,0.22)	0.18 (0.02,0.35)	0.28 (0.09,0.47)	0.36 (0.12,0.59)	0.42 (0.14,0.70)	0.47 (0.13,0.81)

Table S3: Mutually adjusted effects of the categorical potential confounders on the mean BMI *z*-score and BMI *z*-score quantiles obtained from the final models estimating the effect of maternal smoking during pregnancy (AMMs and AQMMs).

Variable	Gender	Mean	10th quantile	25th quantile	50th quantile	75th quantile	90th quantile
Maternal weight							
overweight	boys	-0.01 (-0.16,0.15)	0.03 (-0.15,0.19)	0.03 (-0.07,0.13)	0.01 (-0.03,0.06)	0.02 (-0.02,0.07)	0.02(-0.04,0.09)
obese	boys	0.01 (-0.23,0.26)	-0.10 (-0.40,0.14)	-0.04 (-0.27,0.14)	0.01(-0.16,0.18)	0.05 (-0.08,0.20)	0.06 (-0.05,0.22)
overweight	girls	0.13 (-0.03,0.28)	0.03 (-0.10,0.17)	0.01 (-0.11,0.10)	0.04 (-0.05,0.12)	0.08 (-0.01,0.16)	0.08 (-0.01,0.16)
obese	girls	0.06 (-0.19,0.31)	-0.18 (-0.69,0.26)	-0.09 (-0.49,0.30)	-0.02 (-0.35,0.29)	0.01 (-0.31,0.32)	0.03 (-0.22,0.25)
Maternal education							
≤ 9 years of school education	boys	0.08 (-0.08,0.23)	-0.10 (-0.22,0.01)	-0.05 (-0.12,0.02)	-0.02 (-0.08,0.04)	-0.01 (-0.06,0.05)	0.00(-0.05,0.05)
10-12 years of school education	boys	0.02 (-0.12,0.15)	-0.01 (-0.09,0.08)	0.00 (-0.07,0.08)	0.03 (0.00,0.08)	0.07 (0.02,0.13)	0.08 (0.02,0.17)
≤ 9 years of school education	girls	0.12 (-0.04,0.27)	0.01 (-0.16,0.19)	0.03 (-0.11,0.19)	0.04 (-0.05,0.15)	0.05 (-0.02,0.18)	0.05 (-0.01,0.14)
10-12 years of school education	girls	0.07 (-0.07,0.21)	-0.09 (-0.18,0.03)	-0.02 (-0.09,0.07)	0.03 (-0.03,0.11)	0.10 (0.04,0.16)	0.13 (0.04,0.22)
Gestational age							
small	boys	-0.49 (-0.68,-0.30)	0.00 (-0.07,0.07)	0.01 (-0.03,0.08)	0.02 (-0.02,0.08)	0.04 (-0.02,0.09)	0.06 (-0.02,0.17)
large	boys	0.73 (0.51,0.95)	-0.13 (-0.39,0.08)	-0.08 (-0.26,0.07)	-0.03 (-0.16,0.08)	0.01 (-0.10,0.09)	0.02 (-0.05,0.11)
small	girls	-0.69 (-0.87,-0.51)	0.03 (-0.11,0.15)	0.06 (-0.05,0.16)	0.10 (0.01,0.18)	0.14 (0.04,0.23)	0.14 (0.05,0.22)
large	girls	0.64 (0.43,0.86)	-0.25 (-0.67,0.09)	-0.14 (-0.45,0.14)	-0.08 (-0.32,0.13)	-0.07 (-0.30,0.13)	-0.03 (-0.19,0.12)
Breastfeeding							
no	boys	-0.05 (-0.27,0.17)	-0.05 (-0.13,0.02)	-0.03 (-0.08,0.02)	0.02 (-0.03,0.05)	0.05 (-0.01,0.13)	0.06 (-0.02,0.19)
no	girls	0.16 (-0.06,0.38)	0.00 (-0.17,0.17)	0.05 (-0.07,0.19)	0.05 (-0.03,0.15)	0.10 (0.05,0.17)	0.14 (0.04,0.23)
Preterm delivery							
yes	boys	-0.66 (-1.02,-0.30)	-0.01 (-0.08,0.06)	0.02 (-0.03,0.08)	0.02 (-0.03,0.09)	0.03 (-0.02,0.11)	0.05 (-0.01,0.14)
yes	girls	-0.58 (-0.92,-0.25)	0.01 (-0.15,0.14)	0.05 (-0.07,0.16)	0.08 (0.00,0.18)	0.13 (0.04,0.21)	0.12 (0.05,0.21)
Paternal smoking							
yes	boys	-0.02 (-0.16,0.12)	-0.11 (-0.22,-0.03)	-0.05 (-0.13,0.01)	-0.02 (-0.09,0.02)	-0.01 (-0.07,0.04)	-0.01 (-0.07,0.05)
yes	girls	0.01 (-0.12,0.14)	-0.07 (-0.18,0.05)	-0.05 (-0.15,0.05)	0.00 (-0.07,0.07)	0.04 (-0.02,0.13)	0.05 (0.00,0.13)

Table S4: Univariate and multivariate effect estimates β (95%CI) of the potential confounders only available in either MAS (early adiposity rebound, weight gain during the first two years of life) or KOPS (TV consumption, physical activity) on the mean BMI *z*-score.

Variable	Univariate	Multivariate
TV consumption, >1 hour		
boys	0.35 (0.08,0.63)	0.35[a] (0.07,0.64)
girls	0.45 (0.23,0.68)	0.43[a] (0.20,0.66)
Physical activity in a sports club, ≤2 hour		
boys	0.13 (-0.16,0.41)	0.10[b] (-0.18,0.38)
girls	-0.21 (-0.46,0.04)	-0.20[b] (-0.43,0.04)
Early adiposity rebound		
boys	0.20 (0.03,0.36)	0.09[c] (-0.05,0.24)
girls	0.11 (-0.08,0.30)	0.02[c] (-0.13,0.18)
Weight gain during the first year of life		
boys	0.22 (0.19,0.25)	0.21[d] (0.17,0.24)
girls	0.21 (0.18,0.24)	0.21[d] (0.17,0.24)

[a]Adjusted by the interaction term of maternal smoking during pregnancy and age and by physical activity in a sports club. [b]Adjusted by the interaction term of maternal smoking during pregnancy and age and by TV consumption. [c]Adjusted by the interaction term of maternal smoking during pregnancy and age and by weight gain during the first year of life. [d]Adjusted by the interaction term of maternal smoking during pregnancy and age and by early adiposity rebound.

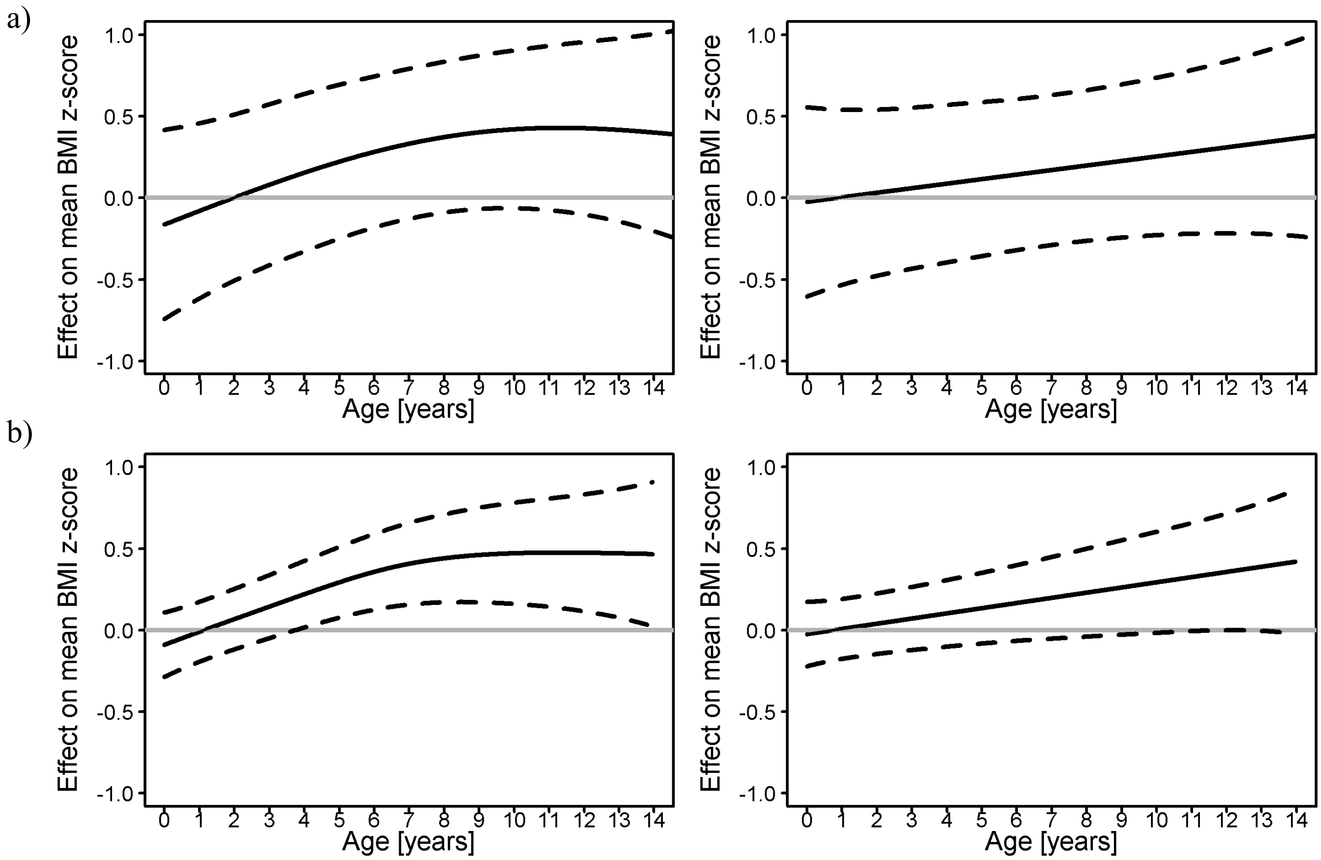

Figure S3: Age-varying effect of maternal smoking during pregnancy (black lines) compared to non-smoking mothers during pregnancy (grey horizontal line at zero) for boys (left panel) and girls (right panel) adjusted by a) TV consumption and physical activity in a sports club (KOPS n = 107 boys and 132 girls) and b) early adiposity rebound and weight gain during the first year of life (MAS n = 351 boys and 299 girls) resulting for mean BMI *z*-score values from the additive mixed model. The black lines show the estimated effect and the dashed lines the 95% CI.
